# Correction to: A remotely delivered, peer-led intervention to improve physical activity and quality of life in younger breast cancer survivors

**DOI:** 10.1007/s10865-022-00390-7

**Published:** 2023-01-18

**Authors:** Lauren S. Weiner, Stori Nagel, H. Irene Su, Samantha Hurst, Susan S. Levy, Elva M. Arredondo, Eric Hekler, Sheri J. Hartman

**Affiliations:** 1 Herbert Wertheim School of Public Health and Human Longevity Science, UC San Diego, La Jolla, CA USA; 2 UC San Diego Moores Cancer Center, 3855 Health Sciences Drive, La Jolla, CA USA; 3 Haus of Volta, Murrieta, CA USA; 4 Division of Reproductive Endocrinology and Infertility, Department of Obstetrics, Gynecology, and Reproductive Sciences, UC San Diego, La Jolla, CA USA; 5 School of Exercise and Nutritional Sciences, San Diego State University, San Diego, CA USA; 6 Institute for Behavioral and Community Health, San Diego State University Research Foundation, San Diego, CA USA; 7 School of Public Health, San Diego State University, San Diego, CA USA; 8 Center for Wireless & Population Health Systems, Qualcomm Institute, San Diego, CA USA

**Correction to: Journal of Behavioral Medicine** 10.1007/s10865-022-00381-8

Due to publisher processing error, the following manuscript sessions were omitted from the original version of this article: Supplemental Material 1, Acknowledgements. Additionally, the original version of this article unfortunately contained errors in Table 4. The omitted sections and corrected Table 4 are given below.

**Table 4:** Physical activity and quality of life outcomes at T0, T1, and T2 and changes between time points among Pink Body Spirit participants

Outcome	N	Time point			Change			Repeated measures ANOVA		
		T0	T1	T2	T0 to T1	T0 to T2	T1 to T2	F value	*p* value	n^2^
		M (SD)	M (SD)	M (SD)	M (SD)	M (SD)	M (SD)			
MVPA^a^	30	90.2 (49.9)	129.9 (84.2)	127.4 (86.8)	+ 39.7 (63.4)	+ 37.2 (76.4)	− 2.5 (71.6)	5.94	< 0.005	0.06
Strength training^b^	32	0.1 (0.3)	0.9* (1.3)	1.1* (1.4)	+ 0.8 (1.2)	+ 1.0 (1.4)	+ 0.2 (1.2)	11.2	< 0.001	0.14
Stretching & flexibility^c^	31	0.23 (0.5)	1.06* (1.6)	1.32* (1.7)	+ 0.8 (1.2)	+ 1.1 (1.5)	+ 0.3 (1.7)	8.46	< 0.001	0.11
Body image^d^	34	17.2 (8.4)	13.2 (8.2)	11.3* (7.1)	− 4.0 (5.7)	− 5.9 (6.1)	− 1.9 (4.1)	21.24	< 0.001	0.09
Sexual function^e,f^	34	14.8 (10.7)	15.2 (11.0)	15.2 (11.9)	+ 0.4 (6.2)	+ 0.1 (5.9)	− 0.1 (7.0)	0.117	< 0.889	0.004
Fatigue^g,h^	33	60.2 (8.2)	54.3* (8.1)	54.0* (9.0)	− 5.8 (8.5)	− 6.1 (8.4)	− 0.3 (7.5)	11.94	< 0.001	0.1
Anxiety^g,i^	33	60.7 (7.6)	57.1 (9.5)	57.0 (9.3)	− 3.6 (9.7)	− 3.7 (7.5)	− 0.1 (9.8)	3.58	< 0.034	0.04
Depression^g^	34	54.0 (9.0)	52.2 (9.2)	51.7 (8.0)	− 1.8 (7.1)	− 2.0 (6.2)	− 0.5 (6.6)	2.18	< 0.121	0.01
Emotional support^g^	34	46.5 (8.2)	49.2 (8.6)	50.8 (9.6)	+ 2.7 (6.4)	+ 4.3 (6.3)	+ 1.6 (6.4)	7.86	< 0.001	0.04

*M* Mean, *SD* Standard Deviation

^a^*MVPA* Moderate to Vigorous Physical Activity measured with ActiGraph accelerometer, min/week, removed 4 outliers due to high MVPA at T0; ^b^days/week, removed 2 outliers with high strength training at T0; ^c^days/week, removed 3 outliers with high stretching & flexibility at T0 or T1; ^d^Score range = 10–40; ^e^score range = 2–36; ^f^Removed 12 participants who were not sexually active at T0; ^g^PROMIS measures reported as standardized t-score with mean = 50 and SD = 10; ^h^Removed 1 outliers with high low fatigue at T0; ^i^Removed 1 outlier with low anxiety at T0; *significantly (*p* < 0.05) different from T0; η^2^ = eta-squared effect size

## Supplementary Information

Below is the link to the electronic supplementary material.

Supplementary file1 (DOCX 16 kb)

